# Family medicine internship support during the COVID-19 pandemic in Cape Town, South Africa – A narrative report

**DOI:** 10.4102/phcfm.v12i1.2661

**Published:** 2020-11-06

**Authors:** Gaironesa Solomon, Ayesha Allie, Raeesah Fakier, Daniel Tadmor, Kamaludin Ashtiker, Colyn Le Roux, Junaid Omar, Mosedi Namane

**Affiliations:** 1 Vanguard Community Health Centre, Metro Health Services, Western Cape Department of Health, Cape Town, South Africa; 2 Division of Family Medicine, Faculty of Health Sciences, University of Cape Town, Cape Town, South Africa

**Keywords:** medical-interns, supervision, COVID-19, pandemic, Cape Town

## Abstract

The health-service redesign that came with the preparation for the surge of COVID-19 had a potential of disrupting the Family Medicine internship programme like it did to many other health and academic programmes. A team of Cape-Town based Community Health Centre (CHC) doctors mitigated this challenge by designing an innovative tool that facilitated ongoing supervision of the interns in order to achieve the outcomes of the Health Professions Council of South Africa (HPCSA).

## Background

In South Africa (SA), internship of newly qualified medical doctors is considered part of medical training. It is a 2-year post that is followed by 1-year community service before doctors can work independently as general practitioners or follow the stream of training further in a specialty of their choice. Both internship and community-service are compulsory and are remunerated. The supervision of SA-medical interns during a Family Medicine rotation is based on the premise that the accredited training platforms have the capacity to expose interns to a full package of primary care services within a community.^1^ This rotation spans 4-months (inclusive of 1 month of psychiatry) within a total of 2-year period of training.^1^ The interns are supervised to acquire core skills such as the management of undifferentiated illnesses. Our Community Health Centre (CHC) is an accredited 24-h urban-based training facility that provides free public-healthcare services to the surrounding communities of poor socio-economic status.

The COVID-19 surge became apparent in SA in March 2020. It necessitated significant service-redesign and a massive de-escalation of ‘non-essential’ services, for the purpose of increasing the capacity to manage patients and to improve infection control.

COVID-19 pandemic not with standing, a cadre of competent primary care clinicians is still going to be required to support the journey of SA towards Healthcare 2030^2^ and Universal Health Coverage.^3^ This is what motivated our team of doctors (authors of this report) to innovate a system that would limit supervision deficiencies as was found by Bola et al.^4^

## Innovation

A team comprising of medical officers (experienced in rendering primary care), community-service doctors (who completed their internship at the end of 2019) and a Family Physician (well-versed in the primary-healthcare approach) participated in the development of the **Va**nguard **S**upervision **T**ool for **M**edical **I**nterns (VaSTMI-COV19) using a modified Delphi approach. This approach is a communication process of experts over multiple rounds of deliberations, which allows them to reach a consensus about a framework they are developing. The participants started the process by examining the Health Professions Council of South Africa (HPCSA) logbook for the desired outcomes of training.^1^ This was followed by several rounds of discussions taking place in the month of April 2020 to refine a table that matched core-objectives of training with required skills and appropriate methods of supervision until a consensus was reached. Infection Prevention Control (IPC) and Occupational Health and Safety (OHS) training and practice were included. The interns were given an opportunity to comment on the contents of the table and where necessary, adjustments were made. May 2020 served as a pilot and implementation month.

**TABLE 1:** Vanguard supervision tool for medical interns during covid-19 pandemic (vastmi-COVID-19).

Supervision method	HPCSA objectives	Skills to be gained
**Orientation** (by clinical manager)	Understand the district health system and its components (facility-based services, home- and community-based care services, healthcare teams, referral pathways, stakeholders, support systems) Staff-safety matters	Understand comprehensive healthcare. Understand continuation of care. Understand continuum of care. Know the community served.
**Teamwork** (Learning conversations)	Management of emergencies.	Reflect on experience Appreciate own role in the team. Understand clinical forensic medicine at the primary level.
**Daily ‘opportunistic’ teaching** (by attending supervisor)	Management of ‘walk-in’ patients. Rational prescribing of medication. Rational use of investigations.	Management of undifferentiated problems. Learn cost-effective approach. Writing of legal prescriptions Reporting of adverse-drug reactions.
**Quizzes** (During scheduled times)	Interpretation of ECGs, X-rays and laboratory investigations. Pattern recognition and management of common conditions, for example, eczema.	To integrate skills, knowledge and experiences gained in other domains of internship placement or in prior learning as a medical student.
**Video-based teaching** (Promote remote learning to enhance and complement practical training)	Videos identified include: How to suture. How to insert an intercostal drain. ‘Donning’ and ‘Doffing’ of *PPE.	Gain competence in: common procedural skills. COVID-19 related IPC and OHS.
**Folder-reviews** (performed whilst renewing medicine-scripts of de-escalated patients)	Critique: Management of diabetes, hypertension, asthma/COPD, epilepsy, HIV, TB, common musculoskeletal and mental health conditions. Whether prescribing in accordance with national guidelines.	Gain competence in: Comprehensive management of common chronic conditions. Application of family medicine, palliative care and PHC principles. Integration of public health in care.
**Attend discussions of new policies, circulars and guidelines** (led by senior doctors)	Participate in evolving clinical practice based on new policies, circulars and guidelines.	Evidence-based practice.
**Ethical discussions** (Learning opportunities provided by COVID-19)	Obtaining informed consent. Respect for confidentiality. Respect for the dignity of persons/autonomy. Promote social justice. Withholding or withdrawing treatment.	Develop sensitivity for ethical issues, cultural differences and human rights. Promote ‘living’ the Western Cape Government’s *C^2^AIR^2^ values.
**Role playing** (Led by senior doctor)	Motivational interviewing for behaviour change. Breaking bad news. Holding a family conference.	Develop communication and counselling skills.
**Debriefing following resuscitations** (either post- emergency unit calls or during *M&M meetings)	Approach to: Adult and paediatric resuscitations. Adult cardiac arrest. – A shocked child/convulsing child/child with breathing difficulties. Managing a hypoxic PUI/ COVID-19 patient	Gain competency in emergency-care skills. Learn how to address gaps identified in *M&M meetings. See the value of debriefing on mental health.
**Intern-led seminars** (based on Intern-identified topics)	Example: Contraception methods.	Promote continued professional development. Harness leadership skills
**Folder audits** (use provincial and national audit tools)	Rational use of antibiotics. Record keeping according to national core standards.	Promote: antibiotic stewardship proper documentation auditing

M&M, morbidity & mortality; PUI, person under investigation or suspect for COVID-19; PPE, personal protective equipment; C^2^AIR^2^, caring, competence, accountability, integrity, responsiveness and respect.

## Conclusion

Feedback from interns suggests that this alternative supervision method is enjoyable, promotes supportive relations between interns and supervisors and creates opportunities to learn from each other. Regarding supervisors, we observed some of their previously unknown strengths emerging organically during VaSTMI-COV19 led processes, with one colleague, for example, ending-up as a ‘natural’ training co-ordinator and another as an information-technology ‘consultant’. The Covid-19 pandemic has afforded all healthcare workers to learn and ‘figure out’ new things together that are related to the pandemic, and the training tool facilitated that interns be part of that experience. A formal qualitative study will be required to assess whether this tool is able to produce ‘good interns’ with attributes described by De Villiers et al.^5^ We are hopeful that this tool will serve as a template that can be adjusted by others for use in their setting during this pandemic and perhaps beyond.
